# Visual Field Loss Progression after Macular Hole Surgery

**DOI:** 10.1155/2009/617891

**Published:** 2010-01-13

**Authors:** Gian Marco Tosi, Gianluca Martone, Angelo Balestrazzi, Alex Malandrini, Marco Alegente, Patrizia Pichierri

**Affiliations:** Department of Ophthalmology and Neurosurgery, University of Siena, 53100 Siena, Italy

## Abstract

*Purpose*. To report a patient who experienced visual field loss progression after vitrectomy for an idiopathic stage II macular hole. *Methods*. Case report. A 68-year-old woman, with no history of glaucoma or any neuroophthalmological diseases, underwent a vitrectomy for a macular hole. *Results*. The patient showed macular hole closure and a resulting central visual acuity of 20/20. However, two months after surgery, she developed an inferotemporal visual field defect. Moreover, seven months after surgery, the patient noticed an enlargement of the temporal blind area: a nearly complete temporal defect was confirmed on visual field testing. *Conclusions*. Although the beneficial results of successfully treated macular holes are unquestionable, this report raises the possibility that visual field defects following macular hole surgery may be progressive.

## 1. Introduction

Visual field defects after pars plana vitrectomy for a full thickness, idiopathic macular hole were first documented in 1995 by Melberg and Thomas [1]. Since then, this complication has been reported by a number of authors who have described peripheral visual field loss that occurred after disappearance of the gas bubble and remained unchanged during the follow-up period [1–7].

We describe a woman who, two months after surgery, developed an inferotemporal visual field defect following pars plana vitrectomy for an idiopathic macular hole. Seven months after surgery, she presented an enlargement of the temporal blind area: a nearly complete temporal defect was documented on visual field testing.

## 2. Case Report

A 68-year-old woman complained of blurred vision and metamorphopsia in her left eye (LE) for three months. Her ocular history was otherwise unremarkable and her general history was positive for hypertension and allergic asthma. Visual acuity was 20/20 in her right eye (RE) and 20/200 in her LE.

Anterior segment examination showed a moderately shallow anterior chamber and cortical cataract in both eyes. Intraocular pressure (IOP) was 25 mmHg in her RE and 24 mmHg in her LE.

Fundus examination of her RE was normal while the left fundus showed a stage II macular hole, confirmed by ocular coherence tomography (OCT).

She underwent cataract surgery and pars plana vitrectomy in her LE. Briefly, under peribulbar anesthesia, phacoemulsification and intraocular lens implantation were performed; this was followed by standard three-port pars plana vitrectomy with the infusion cannula placed inferotemporally. After a core vitrectomy, posterior vitreous detachment was surgically induced using the vitreous cutter with active aspiration over the optic disk and then extended outward to the equator. The vitrectomy was completed, with the vitreous gel removed as far out toward the periphery as possible. No removal of the epiretinal membrane or the internal limiting membrane was performed. Neither papillary nor peripapillary hemorrhages were noted intraoperatively by the surgeon or assistant. 

Fluid/air exchange was performed by passive aspiration using a backflush needle over the optic disk. During fluid/air exchange, the air pressure was set at 30 mmHg. No air humidifier was used as part of the air infusion system. No direct trauma of the disk or the peripapillary area was observed by the surgeon or assistant during the procedure. 

After superior sclerotomies were closed with 7-0 Vycril sutures, an air/gas exchange was performed by injecting 12% perfluoropropane (C_3_F_8_) with a 20 cc syringe through the superonasal sclerotomy while the air infusion line was cut and left open. After the syringe was flushed, the infusion line was clamped. The inferotemporal sclerotomy was then closed with a 6-0 Vycril suture. 

The patient was asked to maintain a face-down position for 15 days. 

On the first postoperative day, vision was hand motions; an IOP of 40 mmHg was noted but returned to normal within a few days after topical aqueous suppressants and 500 mg oral acetazolamide were administered. Fundus examination showed an 80% gas bubble present and, although the view was partially obscured by the gas bubble, no signs suggestive of optic disk or retinal disease were observed. 

Follow-up examinations at seven days, 15 days, and 30 days after surgery showed a gradual improvement of visual acuity from hand motions to 20/30, an IOP within normal range under topical aqueous suppressants and normal fundus appearance together with a closed macular hole (30 days after surgery), confirmed by OCT. 

**Figure 1:**
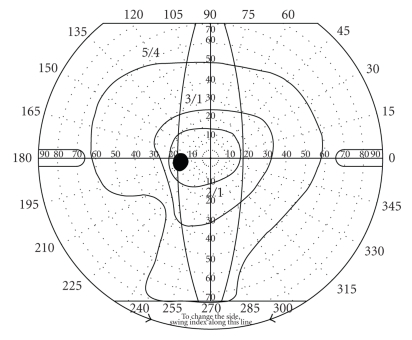
Goldmann visual field test two months after surgery revealed an inferotemporal visual field defect in the left eye.

Two months postoperatively, after the gas bubble was completely reabsorbed, the patient noticed a blind area in the inferotemporal visual field of her LE. She was immediately examined, showing an improvement of visual acuity to 20/25, a controlled IOP, and normal fundus examination. Goldmann perimetry confirmed the patient's symptoms, revealing an inferotemporal visual field defect (Figure 1). We examined the patient again three months after surgery and no changes were observed; the patient was relatively happy and she was getting used to the inferotemporal blind spot. The remaining postoperative controls were carried out by the referring ophthalmologist. 

**Figure 2:**
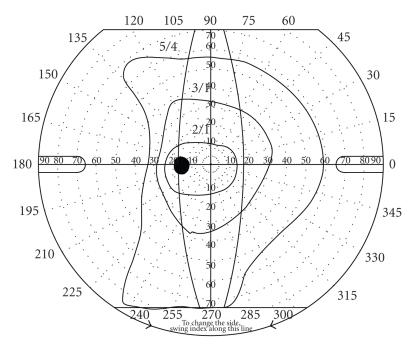
Goldmann visual field test seven months after surgery revealed a nearly complete temporal visual field defect in the left eye.

However, seven months after surgery, the patient noticed an enlargement of the temporal blind area. She was immediately examined, showing an improvement of visual acuity to 20/20, a controlled IOP, and a normal fundus examination. A nearly complete temporal defect was confirmed on visual field testing with Goldmann perimetry (Figure 2). 

Neurological examination and magnetic resonance imaging of the head were performed and did not reveal any abnormalities.

Sixteen months after surgery, the patient's condition is unmodified and, although happy about the recovery of her central visual acuity, she still feels disappointed about her visual field defect. 

## 3. Discussion

The exact mechanism of visual field loss after macular hole surgery remains unclear. 

Papillary and peripapillary tractions during cortical vitreous peeling, phototoxic effects from exposure to the intraocular fiberoptic illuminators, direct mechanical trauma to the optic disk, fluid/air exchange (infusion cannula positioning, high air pressure, and dehydration injury of the retinal nerve fiber layer), mechanical or toxic effects of the gas bubble, retinal or ciliary artery occlusion, and glaucomatous damage from elevated postoperative IOP are among the factors implicated in the pathogenesis of peripheral visual field loss [3]. 

In our opinion, what happened in the present case does not contribute to an understanding of the pathogenesis of peripheral visual field loss after macular hole surgery, but, on the contrary, raises many unanswered questions. Perhaps the extensive description of the surgical procedure performed will help readers find an explanation as to why the visual field defect occurred and why it progressed.

In particular, combining cataract surgery with macular hole surgery is unlikely to be the cause of visual field defect; Ohji et al. [7] found a significantly lower incidence of visual field defects with the combined procedure than in the cases where vitrectomy alone was performed. 

Traction on the peripapillary region during posterior vitreous peeling could potentially have caused shearing damage to the optic nerve head, retinal arterioles, or nerve fiber layer. However, Gass et al. [3] analyzed 105 eyes undergoing macular hole surgery with complete posterior vitreous peeling and found a less than 1% incidence of peripheral visual field defects. Moreover, postoperative visual field defects have also been reported in stage IV macular holes with an already detached vitreous. 

In the present case, the mobilization and elevation of the posterior vitreous cortex was carried out very carefully with active aspiration without touching the optic nerve head or the peripapillary retina; the posterior cortical vitreous was easily peeled without noting any papillary or peripapillary hemorrhages during the procedure. 

Mechanical damage by air infusion on the fundus area located controlaterally to the infusion cannula is another mechanism implicated in peripheral visual field defects after macular hole surgery [3]. Hirata et al. [2] found a significantly lower incidence of peripheral visual field defects in patients with infusion air pressure set at 30 mmHg compared to 50 mmHg (air was humidified in both groups). Dehydration injury to the retina is another phase of fluid/air exchange implicated in visual field defects after macular hole surgery. Ohji et al. [7] observed that passing air through water before injection into the eye significantly reduced the incidence of visual field defect after macular hole surgery. However, many authors seem to correlate the reduced incidence of visual field defects more with the low setting of air pressure than with air hydration [2, 3]. In fact, Gass et al. [3] found a less than 1% incidence of visual field defects although the air was not humidified. 

In the present case, the visual field defect might be related to the air flow directed to the superonasal retina. We did not humidify the air before injecting it into the eye, but air perfusion pressure was set at 30 mmHg and, although the visibility near the disk is slightly impaired during fluid/air exchange, we did not notice any trauma of the disk or the peripapillary area. Moreover, we did not observe any retinal abnormalities either during the immediate postoperative period or during the rest of follow-up. 

Some authors have postulated that the gas bubble itself might exert a direct trauma on the retinal tissue, but in the present case the patient's compliance with respect to the face-down positioning was excellent, thus reducing the pressure of the gas bubble on areas different from the macula [2, 6]. 

Since we did not perform any fluorescein angiography in this case, we are unable to exclude retinal or choroidal vascular compromise as a causative agent of the visual field defect. However, upon fundus examination, no signs of vascular disease were noted during the follow-up period. Moreover, the IOP spike during the immediate postoperative period, which was controlled within a few days, is unlikely to be the cause of peripheral visual field loss. In fact, Bopp et al. [6] found no relation between IOP spikes postoperatively and peripheral visual field defects. 

In the present case, the reason why visual field defect occurred remains unclear and it is even more difficult to explain why, seven months after surgery, the patient reported visual field loss progression in the absence of any neurological abnormalities. We can speculate that the high, long-lasting preoperative IOP might have increased the susceptibility of the eye to the insult caused by the surgical procedure and that the patient might have realized the exact extent of the visual field defect only after central visual acuity had improved. However, the improvement of central visual acuity in the months following vitreous surgery should be common to all the other reported cases of visual field defect after macular hole surgery, which, on the contrary, did not present visual field defect progression.

The authors have no proprietary interest in any of the materials used in this study. None of the authors has a financial support for the study.
